# Intraoperative Assessment of Coronary Resistances: A New Quality Marker and Potential Tool to Predict Early Graft Failure after Coronary Artery Bypass Grafting?

**DOI:** 10.3390/jcdd8120163

**Published:** 2021-11-26

**Authors:** Antonino Salvatore Rubino, Fabrizio Ceresa, Liborio Mammana, Giuseppe Vite, Gianluca Cullurà, Augusto Palermo, Aurora Leonardi, Bruna Filomena De Donno, Francesco Patanè

**Affiliations:** Division of Cardiac Surgery, Department of Cardio-Thoraco-Vascular Surgery, Papardo Hospital, 98158 Messina, Italy; fabrizioceresa@aopapardo.it (F.C.); liboriomammana@aopapardo.it (L.M.); giuseppevite@aopapardo.it (G.V.); gianlucacullura@aopapardo.it (G.C.); augustopalermo@aopapardo.it (A.P.); auroraleonardi@aopapardo.it (A.L.); brunadedonno@aopapardo.it (B.F.D.D.); francescopatane@aopapardo.it (F.P.)

**Keywords:** coronary artery bypass graft, coronary resistances, transit-time flowmetry

## Abstract

Intraoperative assessment of graft patency is pivotal for successful coronary revascularization. In the present study we aimed to propose a new, easy to perform tool to assess anastomotic quality intraoperatively, and to investigate its potential reliability in predicting early graft failure. Intraoperative graft flowmetry of 63 consecutive patients undergoing CABG were prospectively collected. Transit time flowmetry and its derivatives were recorded. Coronary resistances were calculated according to Hagen–Poiseuille equation both during cardioplegic arrest and after withdrawal from cardiopulmonary bypass. Angiographic evidence of graft occlusion at follow-up was cross-checked with intraoperative recordings. After a mean follow-up of 10.4 ± 6.0 months, 22 grafts were studied, and occlusion was documented in five (22.7%). Occluded grafts showed lower flows and higher resistances recorded during aortic cross-clamping. Coronary resistances, recorded during aortic cross-clamping, greater than 2.0 mmHg/mL/min, showed a sensitivity of 80% and a specificity of 100% to predict graft failure. We propose the routine recording of coronary resistances during aortic cross-clamping as an additional tool to overcome the acknowledged limitation of TTF to predict graft occlusion at 1 year.

## 1. Introduction

Intraoperative assessment of graft patency is pivotal for successful coronary revascularization. As early graft failure is usually consequential to technical faults (e.g., graft twisting, stretching, kinking or anastomotic errors), current guidelines suggest transit-time flow (TTF) measurements to assess intraoperative graft patency [1].

Several studies have aimed to discriminate the most reliable cut-off for the many available TTF derived parameters, but no one has demonstrated a clear reliability to predict quality and long-term graft patency [2,3,4].

In 1999, Belboul and colleagues demonstrated that coronary vascular resistances can be calculated intraoperatively using TTF [5]. This methodology has already been validated, also employing IABP [6].

In the present study we aimed to propose a new, easy to perform tool to assess anastomotic quality intraoperatively and to investigate its potential reliability in predicting early graft failure.

## 2. Materials and Methods

### 2.1. Study Population and Inclusion Criteria

This is a retrospective analysis of prospectively collected data. From December 2019 to April 2020, intraoperative graft flowmetry of all consecutive grafts were collected in an institutional ad-hoc database. Surgery has always been performed by the same experienced surgeons (FP, FC) on normothermic cardiopulmonary bypass with aortic cross-clamping an intermittent hyperkaliemic blood cardioplegia. Inclusion criteria for the present analysis were age ≥18 years, on pump surgery with aortic cross-clamp, first-time surgery. Patients undergoing off-pump coronary revascularization, emergency procedures and in-hospital deaths were excluded. Sixty-four patients fulfilled the inclusion criteria. One patient (1.6%) died of pneumonia during hospitalization and was thus excluded from the study. Accordingly, 63 consecutive patients were part of the present analysis, accounting for a total of 113 grafts. Informed consent for the operation and for subsequent follow-up for research purposes was obtained from each patient upon hospital admission. The study protocol conforms to the ethical guidelines of the 1975 Declaration of Helsinki.

### 2.2. Graft Flowmetry and Coronary Resistances

Graft function was assessed under stable hemodynamic conditions, generally at 30 min after protamine administration. Flowmetry of the grafts was performed with a transit-time flowmeter (Optima flow QC, Transonic Systems Inc., Ithaca, NY, USA). Different probe sizes (1.5, 2.0, 2.5 mm) were available to avoid distortion or compression of grafts. Mean flow (mL/min) and pulsatility index (PI) were obtained directly from the flowmeter. Mean arterial and central venous pressures were recorded as well.

**Figure 1:**
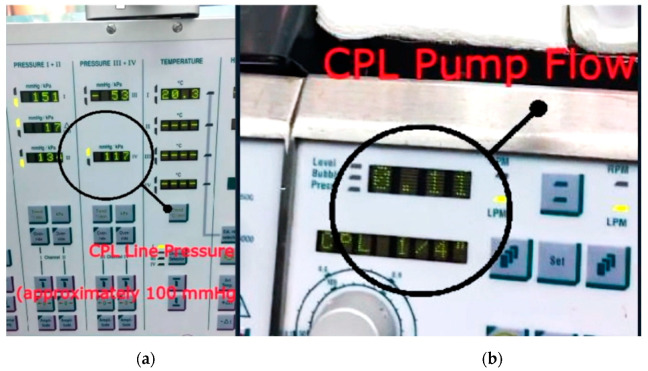
Examples of parameter estimates derived from cardiopulmonary bypass machine: (**a**) pressure drop on the cardioplegia line during selective administration in a venous graft; (**b**) amount of flow selectively delivered.

For the specific aim of this study, flow (mL/min) and pressure drop on the cardioplegic line during selective cardioplegia administration in the venous grafts has been recorded; for homogeneity of data collection, flows were recorded at a standardized pressure of 100 mmHg (Figure 1). Left internal mammary artery to left anterior descending (LIMA-LAD) graft TTFs were also recorded during aortic cross-clamp.

Coronary resistances (CR) were calculated according to the Hagen–Poiseuille equation as CR = (P2 − P1)/Q.

When CR were calculated after withdrawal from cardiopulmonary bypass, P2 is the mean arterial pressure in mmHg, P1 is the central venous pressure in mmHg, and Q is the mean flow (in mL/min) through the graft calculated during TTF analysis [5]. Transit-time flow (TTF) measurements were interpreted as previously reported [7].

When CR are calculated during aortic cross-clamping, differences should be made between venous and arterial grafts. Indeed, for venous grafts, P2 is the pressure drop in the cardioplegic line and Q is the flow of cardioplegia administered selectively in that graft; in the case of mammary artery grafts, P2 is the mean systemic perfusion pressure and Q is the mean flow recorded with TTF probes. In all these cases, P1 is considered equal to 0, as all venous blood return is drained to the reservoir [5].

Angiographic data retrieved from patients undergoing coronary angiography during the first postoperative year, for onset of angina-like symptoms or instrumental signs of ischemia, were cross-checked with intraoperative flow recordings. A FizzGibbon grade greater than A was coded as angiographic occlusion.

### 2.3. Endpoints of the Study

The primary endpoints of this study were the assessment of the safety and technical feasibility to record intraoperative graft flow-derived parameters during aortic cross-clamp, as an alternative tool to beating heart transit-time flowmetry. The secondary endpoint was the identification of appropriate cut-off values to predict early graft failure during follow-up.

### 2.4. Statistical Analyses

Continuous variables are expressed as means and standard deviation, whereas categorical variables are reported as counts and percentages.

Differences in flow-derived parameters between occluded and patent grafts were assessed with an independent sample *t*-test, Mann–Whitney U test and chi-square test.

The univariate association between CR-cardioplegia, CR-protamine, mean flow (included in the model either as a continuous variable or with predetermined cut-offs of <20 mL/min and <31 mL/min according to Queen [2] and Une [8], respectively) and pulsatility index (PI) >3 [2,9] with the angiographic evidence of graft occlusion at follow-up was investigated with logistic regression analysis. In case of quasi-complete separation, exact odds ratios and 95% confidence intervals have been computed.

The discriminant ability of the above-mentioned parameters was assessed, estimating the area under the receiver operating characteristic curve (AUC). An AUC ≥0.8 was considered acceptable. For the specific purpose of our analysis, the DeLong test was used to determine if the differences between the AUCs were statistically significant from CR-cardioplegia [10]. The Youden index was used to identify the best cut-off values. The Bootstrap method with 10,000 resampling was implemented to compute 95% confidence intervals cut-offs, sensitivity, specificity [11].

All statistical analyses were performed using SAS version 9.4 statistical software (SAS Institute, Cary, NC, USA) and R version 3.6.1 (The R Foundation for Statistical Computing, Vienna, Austria) [12] with the cutpointr (version 1.0.1) package. Statistical significance was set at an alpha level of 0.05.

## 3. Results

Baseline characteristics are listed in Table 1.

### 3.1. Intraoperative Flow Measurements

The mean number of grafts per patient was 2.3 ± 1.2. Flow-derived parameters, stratified per graft type and grafted territory are better described in Table 2. As a general rule of thumb, the equipoise between flows and pressures has been considered a quality marker of the coronary anastomosis (e.g., ≥100 mL/min at 100 mmHg for venous grafts [5]; a mean TTF flow at least equal to perfusion pressure for mammary artery grafts).

When the primary safety endpoint is considered, we did not observe any intraoperative adverse event related to the selective administration of cardioplegia in the venous grafts. When the feasibility endpoint is concerned, TTF has been routinely applied to all mammary artery grafts during cross-clamping time, with recordings coherent to what has been observed in case of venous grafts.

### 3.2. Follow-Up Results

After a mean follow-up of 10.4 ± 6.0 months, two patients died (3.2%) (one for stroke and one for COVID-19-related complications after 7 and 13 months, respectively), 51 were asymptomatic and 10 underwent coronary angiography for onset of angina-like symptoms or instrumental signs of ischemia. Overall, 22 grafts were studied, and occlusion was documented in five (22.7%—4 saphenous vein grafts and 1 LIMA graft).

**Figure 2:**
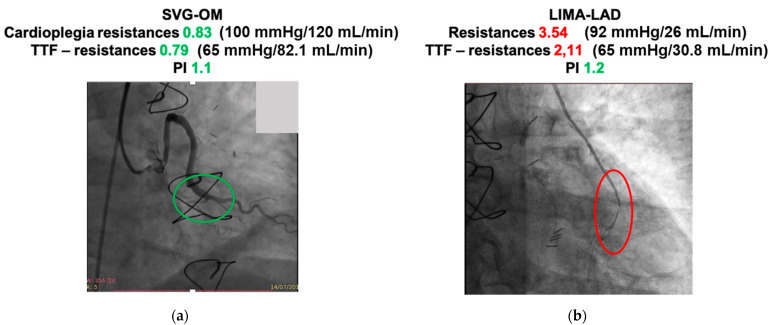
Examples of coronary angiographic evidence combined with intraoperative flowmetric details (**a**) patent graft with satisfactory parameters; (**b**) occluded graft with suboptimal flowmetric values. SVG-OM (saphenous vein graft to obtuse marginal); LIMA-LAD (left internal mammary artery to left anterior descending).

When intraoperative flow measurements were retrieved, occluded grafts showed lower flows and higher resistances recorded during aortic cross-clamping, whereas negligible differences could be observed among TTF-derived parameters recorded after withdrawal from CPB (Table 3 and Figure 2).

For the univariate logistic regression analysis, only CR-cardioplegia was significantly associated with graft occlusion, (exact OR 25.1, exact 95% CI 2.06–> 999.99, *p* = 0.0014) with none of the other recorded or derived parameters reaching statistical significance (Table 4). Interestingly, the risk of graft occlusion increases by 3.8 fold per each 0.5 unit step of CR-cardioplegia.

### 3.3. Discriminant Analysis

**Figure 3:**
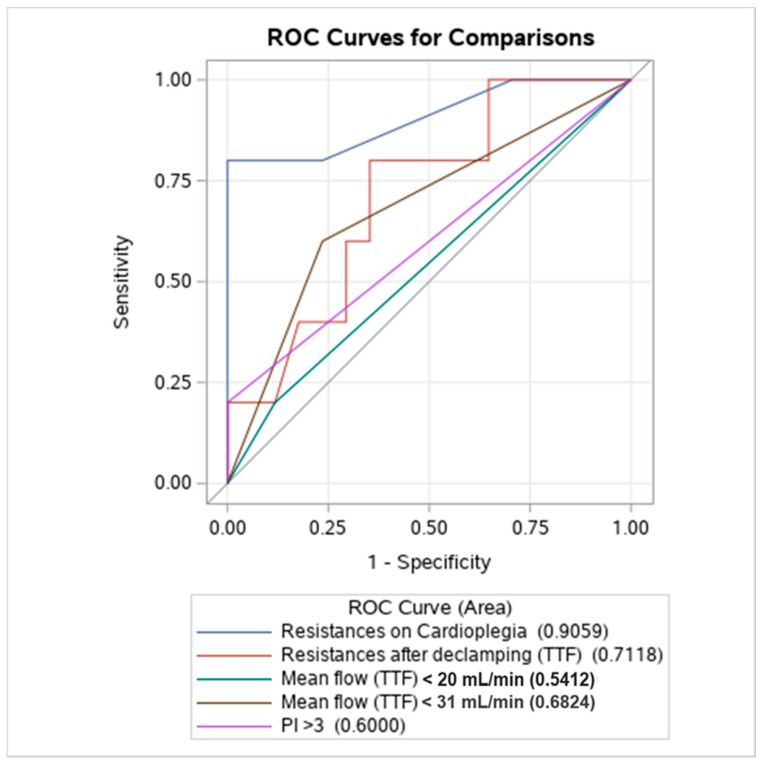
Discriminant ability of different parameters to predict early graft failure. TTF: transit time flowmetry; PI: pulsatility index. ROC (receiver operative characteristic).

ROC analysis showed that only CR-cardioplegia (AUC 0.9059) demonstrated an adequate discriminatory ability for the prediction of graft failure (Figure 3), being higher than any other compared variable (DeLong test vs. CR-protamine *p* = 0.0386; vs. TTF < 20 mL/min *p* = 0.0044; vs. TTF < 31 mL/min *p* = 0.0449; vs. PI > 3 *p* = 0.0110). We identified 2.0 mmHg/mL/min as the most appropriate cut-off, with a sensitivity of 80% and a specificity of 100% (Table 5).

## 4. Discussion

In the present study we observed that coronary resistances can be easily assessed intraoperatively, and can provide useful adjunctive knowledge on the anastomotic graft quality, beside already established technologies, such as TTF. Furthermore, intraoperative assessment of coronary resistances, calculated during aortic cross-clamping time, may predict graft patency at mid-term follow-up.

Intraoperative assessment of graft patency during CABG is mandatory, as it allows the detection and correction of any graft failure. Among all currently available techniques, TTF is one of the most applied methods. Although its use is supported by current guidelines [1], several authors questioned the reliability of flowmetry and its interpretation, which necessitates the complementary evaluation of flows, flow waveform and its derivatives. Accordingly, D’Ancona and coworkers observed that mean flow alone is not sufficiently reliable, and support its coupling with PI [13]. A general agreement does not exist even on the most reliable cut offs to detect graft failure [14]. Accordingly, Nakajima et al. proposed a mean flow <20 mL/min [4], Balacumaraswami considered 5 mL/min unsatisfactory [15]. In the ROOBY trial, TTF < 20 and PI ≥ 3 were considered index of low-flow grafts with poor pulsatility. Finally, the GRIIP trial initially used a mean flow of 10 mL/min as a cut-off for graft failure [16], but a later retrospective revision identified 31 mL/min as a better value [8]. Thus, the absence of a unique interpretation of flow-derived parameters warrants the development of complementary tools. According to the methodology proposed by Belboul and coworkers [5], CR can be easily assessed intraoperatively. Interestingly, CR-cardioplegia recorded in our study were lower compared to those reported by Belboul, even when stratified according to the grafted territory. Generally, we record CR-cardioplegia at a standardized perfusion pressure of 100 mmHg to reach an aimed flow of at least 100 mL/min, whereas Belboul used a maximal pressure of about 60 mmHg and a target flow of 60 mL/min. Although the desired ratio is 1:1 in both studies, whether the observed differences of CR during cardioplegic arrest are related to the distinct perfusion pressures or cooling temperature (normothermic in our study vs. 15 °C in that of Belboul) is still to be determined.

Investigating the CR during cardioplegic arrest provides several interesting pieces of knowledge for the operating surgeon. Indeed, from a physiological perspective, CR has three components: (1) a basal low resistance in the plegic heart, when vessels are maximally dilated and cannot contract; (2) an added basal resistance when vessels have tone; (3) a supplementary phasic resistance during ventricular contraction [17]. Therefore, an increase in CR in the arrested heart is mainly determined by a technical failure of distal anastomosis and cannot be influenced either by competitive flow or poor distal run-off. Hence, it could be speculated that low CR-cardioplegia might be considered a quality marker of coronary anastomosis, with increased values suggesting graft revision.

Although Belboul and coworkers set the benchmark for the methodology used in the present study, information regarding the late fate of the implanted grafts is missing [5]. To the best of our knowledge, this is the first study recording the coronary resistances beyond a LIMA-LAD anastomosis in the arrested heart and the first also reporting the correlation between coronary resistances and graft failure in the early follow-up. In the randomized ROOBY Trial, Quin and coworkers evidenced how grafts with low intraoperative TTF mean flow (<20 mL/min) and high PI (>3) were more often occluded at 1 year follow-up [2]. However, although such cut-offs presented sufficient specificity (>80%), sensitivity was poor (all < 40%). A more recent subanalysis of the ROOBY Trial proved that intraoperative TTF assessment of graft patency was associated with a lower likelihood of having an occluded graft at 1 year (29% vs. 38% non-TTF, *p* = 0.01) [18]. Conversely, Hol and colleagues could not demonstrate any correlation between flows and PI in a prospective cohort of grafts with angiography performed at a 1-year follow-up [19]. In our study, the retrospective analysis of intraoperative TTF recordings showed similar mean TTF flow or PI between occluded and patent grafts, whereas only flows and resistances recorded during cardioplegia were meaningfully different. Furthermore, CR-cardioplegia showed the highest discriminant ability compared to commonly investigated flow derived parameters. According to our experience, we support routine intraoperative assessment of graft patency, and we believe that adjunctive information, readily available at bedside, might improve the predictability of long-term graft performance. In particular, the discriminant ability of the selected cut-off of 2.0 mmHg/mL/min was superior to other parameters, displaying a sensitivity of 80% and a specificity of 100%.

### Limitations

Our study has several limitations. First, the small number of patients included prevent firm conclusions from being drawn on this topic. Secondly, it has been retrospectively designed. Thirdly, it is not a randomized controlled trial. Finally, some patient-related risk factors (e.g., target vessel size, extension of the disease, adherence to optimal medical therapy) are missing, and thus some intraoperative biases might not have been adequately addressed in our models. Future prospective studies, specifically designed to control all patients with computed tomographic coronary angiography, will allow the inclusion of more patients in the analysis and acquire more details on the predictive power of our index.

Conversely, the single-centre design guarantees the uniformity of data collection and of surgical technique.

## 5. Conclusions

Transit time flowmetry remains the most common method used for the intraoperative assessment of the quality of grafts. Coronary resistances are easy to derive intraoperatively and help to identify graft at potential risk of early failure. We propose the routine recording of coronary resistances during aortic cross-clamping as an additional tool to overcome the acknowledged limitation of TTF to predict graft occlusion at 1 year. LIMA-LAD is not a limitation for measurement during aortic cross-clamping.

## Figures and Tables

**Table 1:** Patients’ characteristics.

Baseline	*n* = 63
Age, years	65.1 ± 8.9
Female	20 (31.7)
Hypertension	43 (68.3)
Dyslipidemia	23 (36.5)
Diabetes	29 (46.0)
Peripheral vascular disease	11 (17.5)
COPD	10 (15.9)
Creatinine >200 mmol/L	4 (6.3)
Dialysis	1 (1.6)
Prior myocardial infarction	21 (33.3)
Left main stem	39 (61.9)
LVEF	
>50%	29 (46.0)
30–50%	31 (50.8)
<30%	2 (3.2)
**Intraoperative Details**	
Cardiopulmonary bypass time, min	104.4 ± 42.2
Aortic cross-clamping time, min	88.5 ± 33.0

COPD: chronic obstructive pulmonary disease; LVEF: left ventricular ejection fraction.

**Table 2:** Graft flowmetry and its derivatives stratified according to graft type and coronary territory.

Flowmetry	Graft Type
Parameter	SVG-Right coronary artery (*n* = 6)
Flow during cardioplegia	120.0 ± 31.6
CR cardioplegia	0.84 ± 0.19
Mean TTF flow	47.5 ± 24.0
CR TTF	1.41 ± 0.53
PI	1.4 ± 0.6
Parameter	SVG-Posterior descending artery (*n* = 8)
Flow during cardioplegia	98.8 ± 14.6
CR cardioplegia	1.00 ± 0.20
Mean TTF flow	35.5 ± 19.7
CR TTF	2.83 ± 4.26
PI	1.9 ± 0.9
Parameter	SVG-Obtuse Marginal (*n* = 38)
Flow during cardioplegia	104.5 ± 32.4
CR cardioplegia	1.03 ± 0.30
Mean TTF flow	39.2 ± 21.4
CR TTF	1.94 ± 1.43
PI	1.5 ± 0.5
Parameter	SVG-Ramus intermedius (*n* = 17)
Flow during cardioplegia	118.2 ± 32.6
CR cardioplegia	0.89 ± 0.23
Mean TTF flow	32.7 ± 19.2
CR TTF	2.05 ± 1.38
PI	1.6 ± 0.6
Parameter	SVG-Diagonal (*n* = 17)
Flow during cardioplegia	110.0 ± 26.7
CR cardioplegia	0.92 ± 0.18
Mean TTF flow	29.4 ± 17.8
CR TTF	2.96 ± 2.53
PI	1.8 ± 0.5
Parameter	LIMA-Left anterior descending (20)
Flow during cardioplegia	68.9 ± 33.0
CR cardioplegia	1.33 ± 0.72
Mean TTF flow	49.1 ± 33.4
CR TTF	1.49 ± 0.86
PI	1.6 ± 0.7
Parameter	SVG- Left anterior descending (*n* = 7)
Flow during cardioplegia	110.0 ± 29.4
CR cardioplegia	0.98 ± 0.33
Mean TTF flow	39.5 ± 25.6
CR TTF	1.74 ± 0.88
PI	1.5 ± 0.4

SVG: saphenous vein graft; LIMA: Left internal thoracic artery; CR: coronary resistances, TTF: transit time flowmetry, PI: pulsatility index.

**Table 3:** Differences in graft flowmetry and its derivatives between patent and occluded grafts.

Parameter	Patent Grafts (*n* = 17)	Occluded Grafts (*n* = 5)	*p*
Flow during cardioplegia	100.2 ± 27.7	49.2 ± 31.5	0.0022
CR cardioplegia	1.03 ± 0.33	2.70 ± 1.57	0.0029
Mean TTF flow	54.2 ± 25.5	30.2 ± 17.1	0.06
CR TTF	1.74 ± 0.22	6.38 ± 9.92	0.09
PI	1.7 ± 0.6	2.3 ± 1.6	0.44

CR: coronary resistances, TTF: transit time flowmetry, PI: pulsatility index.

**Table 4:** Univariate logistic regression model to predict graft occlusion.

Parameter	OR	95% CI	*p*
CR cardioplegia	25.1	2.1–>999	0.0420
CR TTF	1.28	0.74–2.20	0.37
TTF < 20 mL/min	1.88	0.13–26.2	0.64
TTF < 31 mL/min	4.88	0.59–40.3	0.14
PI	1.96	0.68–5.64	0.21
PI > 3	11.78	0.11–>999	0.23

CR: coronary resistances, TTF: transit time flowmetry, PI: pulsatility index.

**Table 5:** Bootstrapped cut-off points (with 95% confidence intervals) of transit time flow derived parameters for predictability of 1-year graft occlusion.

Variables	Cut-Off	Sensitivity	Specificity
CR cardioplegia	≥2.0 (2.0–3.5)	80.0% (50.0–100)	100% (100–100)
CR TTF	≥2.03 (1.16–24.1)	80.0% (50.0–100)	64.7% (16.7–100)
Mean TTF flow	≤51.8 (30–51.8)	100% (0–100)	58.8% (33.3–100)

CR: coronary resistances, TTF: transit time flowmetry.

## Data Availability

Not applicable.
